# Multi-locus imprinting disturbance (MLID): interim joint statement for clinical and molecular diagnosis

**DOI:** 10.1186/s13148-024-01713-y

**Published:** 2024-08-01

**Authors:** Deborah J. G. Mackay, Gabriella Gazdagh, David Monk, Frederic Brioude, Eloise Giabicani, Izabela M. Krzyzewska, Jennifer M. Kalish, Saskia M. Maas, Masayo Kagami, Jasmin Beygo, Tiina Kahre, Jair Tenorio-Castano, Laima Ambrozaitytė, Birutė Burnytė, Flavia Cerrato, Justin H. Davies, Giovanni Battista Ferrero, Olga Fjodorova, Africa Manero-Azua, Arrate Pereda, Silvia Russo, Pierpaola Tannorella, Karen I. Temple, Katrin Õunap, Andrea Riccio, Guiomar Perez de Nanclares, Eamonn R. Maher, Pablo Lapunzina, Irène Netchine, Thomas Eggermann, Jet Bliek, Zeynep Tümer

**Affiliations:** 1 Faculty of Medicine, University of Southampton, Southampton, UK; 2 Wessex Clinical Genetics Service, Princess Anne Hospital, University Hospital Southampton NHS Trust, Southampton, UK; 3 Biomedical Research Centre, School of Biological Sciences, University of East Anglia, Norwich Research Park, Norwich, UK; 4 Centre de Recherche Saint Antoine, Endocrinologie Moléculaire et Pathologies d’empreinte, INSERMSorbonne Université, Hôpital Armand TrousseauAPHP, 75012 Paris, France; 5 Department of Human Genetics, Amsterdam Reproduction and Development Research Institute, Amsterdam UMC, University of Amsterdam, Amsterdam, The Netherlands; 6 Division of Human Genetics and Center for Childhood Cancer Research, Children’s Hospital of Philadelphia, Philadelphia, PA 19104 USA; 7 Departments of Pediatrics and Genetics, Perelman School of Medicine at the University of Pennsylvania, Philadelphia, PA 19104 USA; 8 Department of Molecular Endocrinology, National Research Institute for Child Health and Development, Tokyo, Japan; 9 Institut Für Humangenetik, Universitätsklinikum Essen, Universität Duisburg-Essen, Essen, Germany; 10 Department of Laboratory Genetics, Genetics and Personalized Medicine Clinic, Tartu University Hospital, Tartu, Estonia; 11 Department of Clinical Genetics, Institute of Clinical Medicine, University of Tartu, Tartu, Estonia; 12 CIBERER, Centro de Investigación Biomédica en Red de Enfermedades Raras, Madrid, Spain; 13 Institute of Medical and Molecular Genetics, INGEMM-Idipaz, Madrid, Spain; 14 Department of Human and Medical Genetics, Institute of Biomedical Sciences, Faculty of Medicine, Vilnius University, Vilnius, Lithuania; 15 Department of Environmental Biological and Pharmaceutical Sciences and Technologies (DiSTABiF), Università Degli Studi Della Campania “Luigi Vanvitelli”, Caserta, Italy; 16 Regional Centre for Paediatric Endocrinology, Faculty of Medicine, Southampton Children’s Hospital, University of Southampton, Southampton, UK; 17 Department of Clinical and Biological Science, School of Medicine, Centre for Hemoglobinopathies, AOU San Luigi Gonzaga, University of Turin, Turin, Italy; 18 Rare Diseases Research Group, Molecular (Epi)Genetics Laboratory, Bioaraba Health Research Institute, Araba University Hospital-Txagorritxu, Vitoria-Gasteiz, Araba, Spain; 19 IRCCS Research Laboratory of Medical Cytogenetics and Molecular Genetics, Istituto Auxologico Italiano, Milan, Italy; 20 Department of Clinical Genetics, Genetics and Personalized Medicine Clinic, Tartu University Hospital, Tartu, Estonia; 21 Institute of Genetics and Biophysics (IGB),“Adriano Buzzati‐Traverso”, Consiglio Nazionale Delle Ricerche (CNR), Naples, Italy; 22 Aston Medical School, Aston University, Birmingham, UK; 23 Department of Medical Genetics, University of Cambridge, Cambridge, UK; 24 Institute for Human Genetics and Genome Medicine. Faculty of Medicine, RWTH University Aachen, Aachen, Germany; 25 Department of Clinical Genetics, Kennedy Center, Copenhagen University Hospital - Rigshospitalet, Copenhagen, Denmark; 26 Department of Clinical Medicine, Faculty of Health and Medical Sciences, University of Copenhagen, Copenhagen, Denmark

**Keywords:** Differentially methylated regions, DMR, Multi-locus imprinting disturbance, MLID, Imprinting disorder, Clinical diagnosis, Molecular diagnosis

## Abstract

**Background:**

Imprinting disorders are rare diseases resulting from altered expression of imprinted genes, which exhibit parent-of-origin-specific expression patterns regulated through differential DNA methylation. A subgroup of patients with imprinting disorders have DNA methylation changes at multiple imprinted loci, a condition referred to as multi-locus imprinting disturbance (MLID). MLID is recognised in most but not all imprinting disorders and is also found in individuals with atypical clinical features; the presence of MLID often alters the management or prognosis of the affected person. Some cases of MLID are caused by trans-acting genetic variants, frequently not in the patients but their mothers, which have counselling implications. There is currently no consensus on the definition of MLID, clinical indications prompting testing, molecular procedures and methods for epigenetic and genetic diagnosis, recommendations for laboratory reporting, considerations for counselling, and implications for prognosis and management. The purpose of this study is thus to cover this unmet need.

**Methods:**

A comprehensive literature search was conducted resulting in identification of more than 100 articles which formed the basis of discussions by two working groups focusing on clinical diagnosis (n = 12 members) and molecular testing (n = 19 members). Following eight months of preparations and regular online discussions, the experts from 11 countries compiled the preliminary documentation and determined the questions to be addressed during a face-to-face meeting which was held with the attendance of the experts together with four representatives of patient advocacy organisations.

**Results:**

In light of available evidence and expert consensus, we formulated 16 propositions and 8 recommendations as interim guidance for the clinical and molecular diagnosis of MLID.

**Conclusions:**

MLID is a molecular designation, and for patients with MLID and atypical phenotypes, we propose the alternative term multi-locus imprinting syndrome. Due to the intrinsic variability of MLID, the guidelines underscore the importance of involving experts from various fields to ensure a confident approach to diagnosis, counselling, and care. The authors advocate for global, collaborative efforts in both basic and translational research to tackle numerous crucial questions that currently lack answers, and suggest reconvening within the next 3–5 years to evaluate the research advancements and update this guidance as needed.

**Supplementary Information:**

The online version contains supplementary material available at 10.1186/s13148-024-01713-y.

## Background

### Imprinting disorders

Imprinting disorders (ImpDis) are a group of congenital disorders caused by altered expression of imprinted genes, whose expression is normally restricted by their parent of origin [1, 2]. In each imprinted region, parent-of-origin-restricted expression is regulated by an imprinting centre (IC) that acquires differential epigenetic marking (including differential DNA methylation) in the egg and sperm. After fertilisation, differential epigenetic marking is essentially permanent and almost ubiquitous in somatic tissues, notably in the form of DNA methylation at differentially methylated regions (DMRs) [1, 3]. Differential epigenetic marking often spreads beyond the IC to other genes under its control; this includes distinctive patterns of histone modification and non-coding RNA expression, but most notably, further DMRs [1]. Numerous imprinted loci exist in the human genome; some comprise single genes, while others are clusters of genes whose expression is dependent on parental origin (Table 1).

**Table 1:** Human germline imprinted DMRs

DMR name	Other names frequently used in the literature	Associated ImpDis	Location (Hg38)	No. of CpGs	Type^1^	imprint origin
PPIEL:Ex1-DMR			chr1:39,558,953–39,559,868	39	NC	Oocyte_gDMR
DIRAS3:Ex2-DMR			chr1:68,046,821–68047803	88	NC	Oocyte_gDMR
DIRAS3:TSS-DMR			chr1:68,049,749–68051862	39	NC	Oocyte_gDMR
GPR1-AS:TSS-DMR			chr2:206,202,242–206204721	86	X	Oocyte_gDMR
ZDBF2/GPR1:IG-DMR			chr2:206,249,858–206271820	439	NC	Sperm_gDMR-secondary_DMR
NAP1L5:TSS-DMR			chr4:88,697,032–88698086	57	NC	Oocyte_gDMR
VTRNA2-1:DMR			chr5:136,079,112–136080956	76	X	Oocyte_gDMR
FAM50B:TSS-DMR			chr6:3,848,847–3,850,125	90	NC	Oocyte_gDMR
PLAGL1:alt-TSS-DMR	PLAGL1, ZAC1, 6q24	TNDM	chr6:144,006,940–144008751	143	CA	Oocyte_gDMR
IGF2R:Int2-DMR			chr6:160,005,525–160006529	74	NC	Oocyte_gDMR
WDR27:Int13-DMR			chr6:169,654,407–169655522	58	X	Oocyte_gDMR
GRB10:alt-TSS-DMR	GRB10	MLID (ZFP57); upd[7]mat, SRS	chr7:50,781,028–50783615	171	CA	Oocyte_gDMR
PEG10:TSS-DMR			chr7:94,656,224–94,658,648	119	NC	Oocyte_gDMR
MEST:alt-TSS-DMR	MEST	upd(7)mat, SRS	chr7:130,490,280–130494547	226	CA	Oocyte_gDMR
SVOPL:alt-TSS-DMR			chr7:138,663,372–138,664,324	74	NC	Oocyte_gDMR
HTR5A:TSS-DMR			chr7:155,071,008–155071672	55	NC	Oocyte_gDMR
ERLIN2:Int6-DMR			chr8:37,747,473–37,748,570	37	NC	Oocyte_gDMR
PEG13:TSS-DMR			chr8:140,098,047–140100982	193	NC	Oocyte_gDMR
FANCC:Int1-DMR			chr9:95,313,117–95,313,462	26	NC	Oocyte_gDMR
INPP5F:Int2-DMR			chr10:119,818,533–119,819,215	52	X	Oocyte_gDMR
H19/IGF2:IG-DMR & TSS-DMR	IC1, H19	SRS/BWS	chr11:1,997,581–2,003,510	250	CA*	Sperm_gDMR
IGF2:Ex9-DMR		SRS/BWS	chr11:2,132,760–2133882	63	CA	secondary_DMR
IGF2:alt-TSS-DMR		SRS	chr11:2,147,102–2148538	33	CA	Sperm_gDMR
KCNQ1OT1:TSS-DMR	IC2, KvDMR	BWS	chr11:2,698,717–2,701,029	192	CA	Oocyte_gDMR
RB1:Int2-DMR			chr13:48,318,204–48321627	195	NC	Oocyte_gDMR
MEG3/DLK1:IG-DMR	IG-DMR	TS14/KOS14	chr14:100,809,089–100811721	64	CA	Sperm_gDMR
MEG3:TSS-DMR	MEG3	TS14/KOS14	chr14:100,824,186–100827641	188	CA*	secondary_DMR
MEG8:Int2-DMR			chr14:100,904,403–100905082	43	X	secondary_DMR
MKRN3:TSS-DMR			chr15:23,561,938–23,567,348	109	X	Oocyte_gDMR-secondary_DMR
MAGEL2:TSS-DMR			chr15:23,647,277–23,648,882	51	NC	secondary_DMR
NDN:TSS-DMR			chr15:23,686,303–23687612	108	NC	secondary_DMR
SNRPN:alt-TSS-DMR			chr15:24,823,416–24,824,334	19	NC	secondary_DMR
SNURF:TSS-DMR	SNRPN	PWS/AS	chr15:24,954,856–24,956,829	113	CA	Oocyte_gDMR
IGF1R:Int2-DMR			chr15:98,865,266–98,866,421	55	NC	Oocyte_gDMR
ZNF597:3-DMR			chr16:3,431,800–3432388	29	NC	Oocyte_gDMR
ZNF597:TSS-DMR		upd(16)mat	chr16:3,442,827–3,444,463	76	NC	secondary_DMR
ZNF331:alt-TSS-DMR1			chr19:53,537,255–53,538,958	125	NC	Oocyte_gDMR
ZNF331:alt-TSS-DMR2			chr19:53,553,831–53,555,171	102	NC	Oocyte_gDMR
PEG3:TSS-DMR		MLID (ZFP57)	chr19:56,837,124–56,841,903	221	CA	Oocyte_gDMR
MCTS2P:TSS-DMR			chr20:31,546,859–31,548,130	47	NC	Oocyte_gDMR
NNAT:TSS-DMR			chr20:37,520,201–37522126	135	NC	Oocyte_gDMR
L3MBTL1:alt-TSS-DMR			chr20:43,513,724–43,515,400	84	NC	Oocyte_gDMR
GNAS-NESP:TSS-DMR		PHP	chr20:58,838,983–58,843,557	257	CA	secondary_DMR
GNAS-AS1:TSS-DMR	NESP-AS	PHP, upd(20)mat	chr20:58,850,593–58852978	128	CA	Oocyte_gDMR
GNAS-XL:Ex1-DMR		PHP	chr20:58,853,849–58,856,408	200	CA	Oocyte_gDMR
GNAS-A/B:TSS-DMR	GNAS A/B	PHP, upd(20)mat	chr20:58,888,209–58890146	198	CA*	secondary_DMR
WRB:alt-TSS-DMR			chr21:39,385,583–39,386,350	43	NC	Oocyte_gDMR
SNU13:alt-TSS-DMR			chr22:41,681,769–41,682,869	63	NC	Oocyte_gDMR

Clinically associated (CA) is a pragmatic category of loci currently included in diagnostic tests, including direct causative loci, those for upd(7)mat, and those for stratification of TNDM cases caused by *ZFP57* mutations. The majority of germline (g) imprinted loci not in the CA group but are in the non-clinical (NC) group. Xgermline imprinted loci whose methylation shows further polymorphic, somatic or tissue-specific variation; DNA methylation at these loci is not interpretable in relation to clinical imprinting disorders. In imprinted loci with multiple CA-DMRs, the DMR most important for molecular diagnosis is marked with an asterisk (*)

For 13 imprinted loci, altered expression of genes under imprinted control is associated with clinical disorders [2] which, in a recent retrospective analysis, had a total prevalence of 5.8/100,000 [4]. Imprinting disorders have heterogeneous and overlapping features, which impact growth, development, metabolism and behaviour, and early diagnosis is important to implement appropriate management to optimise clinical outcome. Because normal expression of imprinted genes is restricted by parental origin, imprinting disorders can in principle result from any genetic or epigenetic change that disturbs this restricted pattern of expression or gene function. Causes of imprinting disorders include single-nucleotide variants (SNV) or copy number variants (CNV) of coding or *in-cis* regulatory sequences, segmental or whole-chromosome uniparental disomy (UPD), and imprinting defects altering gene expression, particularly DNA methylation disturbance (gain-of-methylation, GOM or loss-of-methylation, LOM) of imprinted DMRs [2].

### Multi-locus imprinting disturbance (MLID)

A proportion of individuals with imprinting disorders have DNA methylation disturbance not at a single imprinted locus, but at multiple imprinted loci across the genome (reviewed in [5, 6]). This phenomenon is designated multi-locus imprinting disturbance (MLID; previous terms include hypomethylation of imprinted loci: HIL, and multi-locus methylation disturbance: MLMD).

Most of the early studies of MLID were restricted both clinically and molecularly: clinically, because they involved research cohorts of patients with canonical imprinting disorders; molecularly, because they assessed imprinted loci associated with canonical imprinting disorders (e.g. Refs. [7–16])﻿. As the scope of molecular analysis expanded, it became clear that MLID involved loci beyond those clinically associated with imprinting disorders (e.g. Refs. [17–23]) and most recent research studies include essentially all known germline DNA methylated regions (e.g. Refs. 24–30).

Clinically, MLID is heterogeneous: the phenotype of an affected person is not readily predictable from their imprinting disturbance [22, 26, 28, 30–34]. Many people with MLID have clinical features aligning with a canonical ImpDis; some have clinical features partially consistent with more than one ImpDis (e.g. Refs. [35–38]); others have some clinical features not aligning with any canonical imprinting disorder (e.g. Ref. [30, 39–43]). The phenotype of MLID is also likely modified by the somatic mosaicism seen in almost all affected individuals (reviewed in [6]).

MLID is recognised in many but not all clinical imprinting disorders. It is found almost exclusively in patients with LOM of a given clinically relevant imprinted region, and seldom in patients whose ImpDis are caused by protein-coding SNV/CNV, CNV of cis-acting regulatory sequences, or UPD (e.g. Refs. [38, 41, 44]). As a corollary of this, some imprinting disorders are not currently associated with MLID, including those caused by protein-coding variants to imprinted genes, such as central precocious puberty (CPP) or Schaaf–Yang syndrome (SHFYNG), and Mulchandani-Bhoj-Conlin syndrome (MCBS), which is associated only with upd[20]mat. In practice, MLID is detected most frequently in imprinting disorders caused by LOM, such as Beckwith-Wiedemann syndrome (BWS) with LOM of IC2 (imprinting centre 2) and Silver–Russell syndrome (SRS) with LOM of IC1, rather than those caused by GOM, such as Kagami–Ogata syndrome (KOS14) or BWS with IC1 GOM.

Table 2 lists the imprinting disorders and the frequency of MLID. It should be noted that the prevalence of MLID across imprinting disorders is not fully established, because surveys have been done differently for different disorders, the disorders themselves are rare, and individuals whose phenotype diverges from canonical presentations may be under-diagnosed. Accordingly, the prevalence of MLID in different disorders is likely to be higher than currently estimated.

**Table 2:** Imprinting disorders

Disorder	Chr (s)	Prevalence	OMIM [113]	% DNA methylation disturbance	% of MLID in imprinting error cases	References
Beckwith-Wiedemann syndrome	11p15.5	1:10,000–1:80,000	#130,650	50% KCNQ1OT1 TSS-DMR LOM*; 10% H19/IGF2 IG-DMR GOM	12.8% Not described	[100,101]
Silver–Russell syndrome	11p15.5, chr7	1:16,000	#180,860	28.3*–38%	5.1% Individual cases	[75,^84^, ^102^]
Transient neonatal diabetes mellitus type 1	6q24	1:15,000–1:400,000	#601,410	30%*	50%	[103]
Pseudohypoparathyroidism type 1b	20q13.3	nk	#603,233	61%*	(0–38%)	[83]
Temple syndrome	14q32	nk	#616,222	33.8*–58.8%	nk	[22, 104]
Kagami–Ogata syndrome	14q32	nk	#608,140	26.6%	Not described	[105, 106]
Angelman syndrome	15q11.2	1:25,000–40,000	#105,830	2–4%	3%	[107, 108]
Prader–Willi syndrome	15q11.2	1:8,000–1:30,000	#176,270	1%	Rare	[108, 109]
Mulchandani-Bhoj-Conlin syndrome	chr20	nk	#617,352	Not described	Not described	[110–112]

nk, not known

*indicates the imprinting disorders and aetiological groups in whom MLID is observed References here are reviews of individual imprinting disorders. References specifically concerning the frequency of multi-locus imprinting disorder in each ID may be found in Sanchez-Delgado et al. [5], and only reports of MLID post-dating this review are cited in this table

### Genetic causes of MLID

In a proportion of affected people, MLID is caused by pathogenic variants in genes involved in epigenetic modifications during early development (reviewed in [45]). The first recognised example of this was pathogenic variants of *ZFP57*, which encodes a DNA-binding factor required for DNA methylation maintenance at many sites including imprinted regions (reviewed in [46]). Approximately half of transient neonatal diabetes mellitus (TNDM) patients with methylation disturbance at *PLAGL1* have biallelic pathogenic variants in *ZFP57*, and have MLID involving LOM at *PLAGL1, GRB10*, and *PEG3* [47–49]. Some patients have been described with additional loci involved in MLID, associated with an atypically severe phenotype [50, 51]. When TNDM with recessive *ZFP57* variants are found in a patient, and parents are heterozygous for the *ZFP57* variants, subsequent offspring are at 25% risk of inheriting biallelic *ZFP57* variants and presenting with TNDM. Another zinc-finger protein with imprinting regulatory function, *ZNF445*, was homozygously inactivated in a patient with Temple Syndrome and MLID [36, 52]. For a separate group of individuals, MLID is associated with pathogenic variants in their mothers, in ‘maternal effect’ genes including *NLRP2, NLRP5, NLRP7, PADI6*, and *KHDC3L.* (e.g. Refs. [34, 37, 40, 53–58]). These genes are very highly expressed from the maternal genome during oocyte maturation, and the maternal effect protein products appear to play essential roles in the early embryo (reviewed in [59]), a time at which epigenetic reprogramming and embryonic genome activation are essential for the onset of development [60]. Their functions remain uncertain because of the difficulty of functional research in the early embryo, and it is possible that further maternal effect genes remain to be discovered.

The severity of effects on offspring varies markedly, even among offspring of individual mothers, ranging from apparently healthy persons to people with MLID, and nonviable reproductive outcomes including recurrent miscarriage, hydatidiform mole, or apparent infertility (e.g. Refs. [34, 55, 57, 58]).

Maternal effect gene variants have a well-recognised association with reproductive difficulties: females with biallelic inactivation of *NLRP7* or *KHDC3L* experience recurrent hydatidiform mole [61, 62], reviewed in 63). Numerous reports exist of maternal effect variants causing early developmental demise in women undergoing fertility treatment (reviewed in [64]). The overlap between maternal effect gene variants, MLID, and reproductive difficulties remains to be clarified by research that takes into account the family as well as the proband (reviewed in 64, 65).

A further overlap exists between assisted reproductive technology (ART) and imprinting disorders. The association between ART and increased risk of some imprinting disorders is well documented [12, 17, 66–69], and a population-based study of three imprinting disorders, Angelman syndrome (AS), Prader–Willi syndrome (PWS) and BWS, showed a higher prevalence of parental fertility problems and greater maternal age than the general population [70]. At present, published studies in MLID are smaller and have given conflicting findings [10, 12, 15, 17, 66] and further research is needed to determine unequivocally whether either ART is associated with elevated risk for MLID as for other imprinting disorders, and/or that some of the association with ART is in fact an association with parental fertility problems related to underlying genetic predisposition.

### Current challenges with MLID

MLID was first identified and characterised in research studies. However, with the increasing clinical recognition of imprinting disorders, and expanding accessibility of commercial diagnostic assays for imprinting, MLID is being increasingly recognised in diagnostic settings (e.g. Refs. [16, 71, 72]).

Patients, families, and health professionals need consensus on the definition of MLID, clinical indications prompting testing, molecular procedures and methods for epigenetic and genetic diagnosis, recommendations for laboratory reporting, considerations for counselling, and implications for prognosis and management, all of which should be pragmatic and acceptable within the ethical, legal, social, and medical systems of different nations.

However, many features of MLID are still being actively researched and many questions remain unresolved. Because MLID is a subset within imprinting disorders that are themselves rare diseases, there is a need for international collaboration between clinical and molecular researchers, combining resources, particularly patient cohort data, to deliver the excellent translational research that will underpin robust evidence-based guidelines in the future.

This interim joint statement aims to meet these two needs with: (a) Propositions for clinical and molecular diagnosis of MLID, and (b) Recommendations for the research required to enable these interim guidelines to be updated in due course with a full clinical consensus for diagnosis and management of MLID. Propositions and Recommendations are presented throughout this joint statement, and collected in Supplementary Tables 1 and 2, respectively.

## The consensus meeting

In June 2023, a meeting in Tallinn, Estonia, brought together 31 expert clinicians and molecular scientists from eleven countries involved in clinical and molecular diagnosis, management, and research on human imprinting, along with four representatives of patient advocacy organisations (PAO). Many of the participants have previously collaborated within an European Union-funded network on imprinting disorders, EUCID (EU Consortium on Imprinting Disorders) [73]. This meeting, funded by the European Joint Programme on Rare Diseases (EJP RD), aimed to develop a ‘road-map’ of work leading to a consensus statement on diagnosis and management of MLID. Therefore, the objectives of the meeting were to: delineate the current molecular and clinical research of MLID; develop interim best practice guidelines for molecular diagnosis; develop an integrated road-map towards a future consensus statement; and plan the collaborative clinical and molecular studies required to underpin that future consensus. This paper addresses these objectives.

### Method

A comprehensive literature search was conducted using PubMed and the search terms “multi-locus imprinting disturbance” and “multi-locus imprinting disorder”. Additional relevant articles were identified by PubMed searches when supplementary information was necessary. These > 100 articles formed the basis of discussion by two working groups (WG).

The working groups focused on clinical diagnosis (WG1) and molecular testing (WG2), had 12 and 19 members, respectively. During eight months of preparation, regular online discussions between WG members determined the questions to be addressed in the meeting and discussed the progress of preparatory documents for each working group. At the face-to-face meeting, propositions and recommendations were considered by WGs separately and discussed in plenary sessions; PAO representatives participated in WG1. Where published data were unavailable or insufficient, the clinical experiences and opinions of the participants were considered. The minutes of the meeting, with the literature and prepared documentation, were combined into guideline propositions and recommendations for research, which were agreed in an online meeting and subsequently written into this joint statement. All participants read, had the opportunity to amend, and assented to the final form of this statement.

## The definition of the term “MLID”

### MLID as imprinting disturbance

The earliest studies of MLID were focused molecularly on DMRs associated with known imprinting disorders, and clinically on patients with imprinting disturbance at these DMRs. In these studies, an imprinting disturbance at two or more loci was sufficient for a designation of MLID without detailed consideration of the molecular abnormality or its clinical correlations. The acronym MLID was used to refer to either Multi-locus imprinting Disturbance (a molecular definition) or Multi-locus imprinting Disorder (a clinical definition) (e.g. [74], versus [5]). The terms could be used interchangeably or were indistinguishable because of the shared acronym; and the distinction was not critical because MLID was sought primarily in patients who had a clinical imprinting disorder and investigated in imprinted loci known to cause imprinting disorders.

Over recent years, more imprinted regions have been included in diagnostic testing, including some that are not directly implicated in an imprinting disorder but act as a reliable proxy for diagnosis. Examples include imprinted DMRs on chr7, which act as a proxy test for upd[7]mat causing Silver–Russell syndrome (SRS) but are not directly associated with specific symptoms of SRS [75].

Clinical case reports and cohort studies do not currently support clear epigenotype-phenotype correlations between the clinical features of people with MLID, and their severity and location of methylation disturbance. At the present time, MLID cannot be unambiguously identified using clinical criteria alone, nor can the epigenotype of an affected person predict their clinical history and vice versa. Therefore, at the present time, multi-locus imprinting disturbance cannot be primarily a clinical diagnosis.

Multi-Locus Imprinting Disturbance (MLID) denotes a molecular state of affairs where a person has DNA methylation disturbance involving multiple germline imprinted loci. The designation MLID is agnostic about the imprinted loci involved, the nature of the imprinting disturbance, and any clinical consequences. Despite the openness of this definition, it is important to emphasise that MLID is not an epigenetic polymorphism or incidental finding, but has potential clinical consequences for the affected person. Methylation disturbance in MLID generally takes the form of LOM, which is often mosaic.

### A pragmatic diagnostic definition of MLID

We propose a pragmatic division of imprinted DMRs into two groups: those that are currently used in clinical diagnosis (clinically associated or CA-DMRs); and those that are not currently used in clinical diagnosis (non-clinical or NC-DMRs) (Table 3). Of note, these imprinted DMR subtypes relate to DNA from blood, which is the most frequently used diagnostic tissue.

**Table 3:** Subtypes of imprinted DMRs^1^

Name	Description	Loci
Clinically associated DMR^2^	DMR where (epi)genetic changes altering expression are directly associated with a canonical imprinting disorder, or are used as a proxy to detect imprinting disturbance or stratify diagnosis^2^	PLAGL1:alt-TSS-DMR; H19/IGF2:IG-DMR^2^; KCNQ1OT1:TSS-DMR; MEG3/DLK1:IG-DMR^2^; SNURF:TSS-DMR; GNAS-A/B:TSS-DMR; GRB10:alt-TSS-DMR; MEST:alt-TSS-DMR; PEG3:TSS-DMR; GNAS-AS1:TSS-DMR; GNAS-NESP:TSS-DMR; GNAS-XL:Ex1-DMR
Non-clinical DMR	DMR whose imprinting is not currently associated with a clinical phenotype or used in diagnosis	PPIEL:Ex1-DMR; DIRAS3:Ex2-DMR; DIRAS3:TSS-DMR; ZDBF2/GPR1:IG-DMR; NAP1L5:TSS-DMR; FAM50B:TSS-DMR; IGF2R:Int2-DMR; PEG10:TSS-DMR; SVOPL:alt-TSS-DMR; HTR5A:TSS-DMR; ERLIN2:Int6-DMR; PEG13:TSS-DMR; FANCC:Int1-DMR; [H19/IGF2TSS-DMR]; IGF2:alt-TSS-DMR; MEG3:TSS-DMR; MEG8 Int2 DMR; RB1:Int2-DMR; MAGEL2:TSS-DMR; NDN:TSS-DMR; SNRPN alt-TSS DMR; IGF1R:Int2-DMR; ZNF597:TSS-DMR; ZNF597:3-DMR^4^; ZNF331:alt-TSS-DMR1; ZNF331:alt-TSS-DMR2; MCTS2P:TSS-DMR; NNAT:TSS-DMR; L3MBTL1:alt-TSS-DMR; WRB:alt-TSS-DMR; SNU13:alt-TSS-DMR

These imprinted DMR subtypes relate to DNA from blood, which is the most frequently used diagnostic tissue. Non-blood tissues may have different methylation profiles, which should be determined using control cohorts before inclusion in diagnostic protocols. Tissue-specific (e.g. placental), transient, and polymorphically imprinted loci are excluded from this table. Categories are based on current understanding and may change as knowledge changes. 2, Currently available MLPA assays (March 2024) use secondary DMR as proxy [72]

CA-DMRs have established diagnostic utility and can be assayed in many diagnostic laboratories. Non-clinical DMRs cannot be tested in many routine diagnostic laboratory settings, and the clinical significance of MLID involving these DMRs is currently not clear. For the present, we propose that diagnostic testing and reporting should take into consideration CA-DMRs only.

Under this pragmatic division, many loci involved in molecular MLID are not included in current clinical care or laboratory diagnostics but remain at present the subject of research to discover their relevance for management and counselling.

Some loci currently deemed non-clinical are frequently implicated in MLID episignatures or may contribute to the atypical clinical features of people with MLID. Detailed research studies are required to assess atypical presenting features in people with MLID, identify associations between these clinical features and epigenetic signatures of MLID, and assess whether and how such epigenotype-phenotype associations should be incorporated into diagnosis and management. We recommend that the research should be periodically reviewed to assess whether currently NC-DMRs are shown to be clinically associated, and to update the group of CA-DMRs accordingly.

### Exclusions from the definition of MLID

It should be noted that methylation disturbance at multiple imprinted loci is not equivalent to MLID when:

(i) the DMRs involved are in the same imprinting cluster, and are subject to co-ordinated imprinting disturbance (e.g. the 14q32 imprinted cluster);
(ii) DNA methylation disturbance involves contiguous DMRs, or DMRs on the same chromosome or chromosomal segment, when it is secondary to UPD or a CNV (however, MLID in addition to UPD is a documented ultra-rare phenomenon (e.g. Refs. [38, 41]));
(iii) uniparental diploidy is present.

#### Propositions

P1. Multi-locus imprinting disturbance (MLID) is a molecular state of affairs where a person has DNA methylation disturbance involving multiple (non-contiguous) germline imprinted loci.

P2. For clinical reporting, MLID is designated as DNA methylation disturbances at ≥ 2 clinically associated (non-contiguous) DMRs

P3. MLID is not constituted by imprinting disturbances of contiguous DMRs under co-ordinated control, or by apparent imprinting disturbances secondary to UPD or CNV.

#### Recommendation for Research

R1. The group of clinically associated DMRs should be periodically reviewed and updated as necessary, based on ongoing clinical research.

## Clinical considerations in diagnosis of MLID

### First-line or second-line MLID testing

**Fig. 1:**
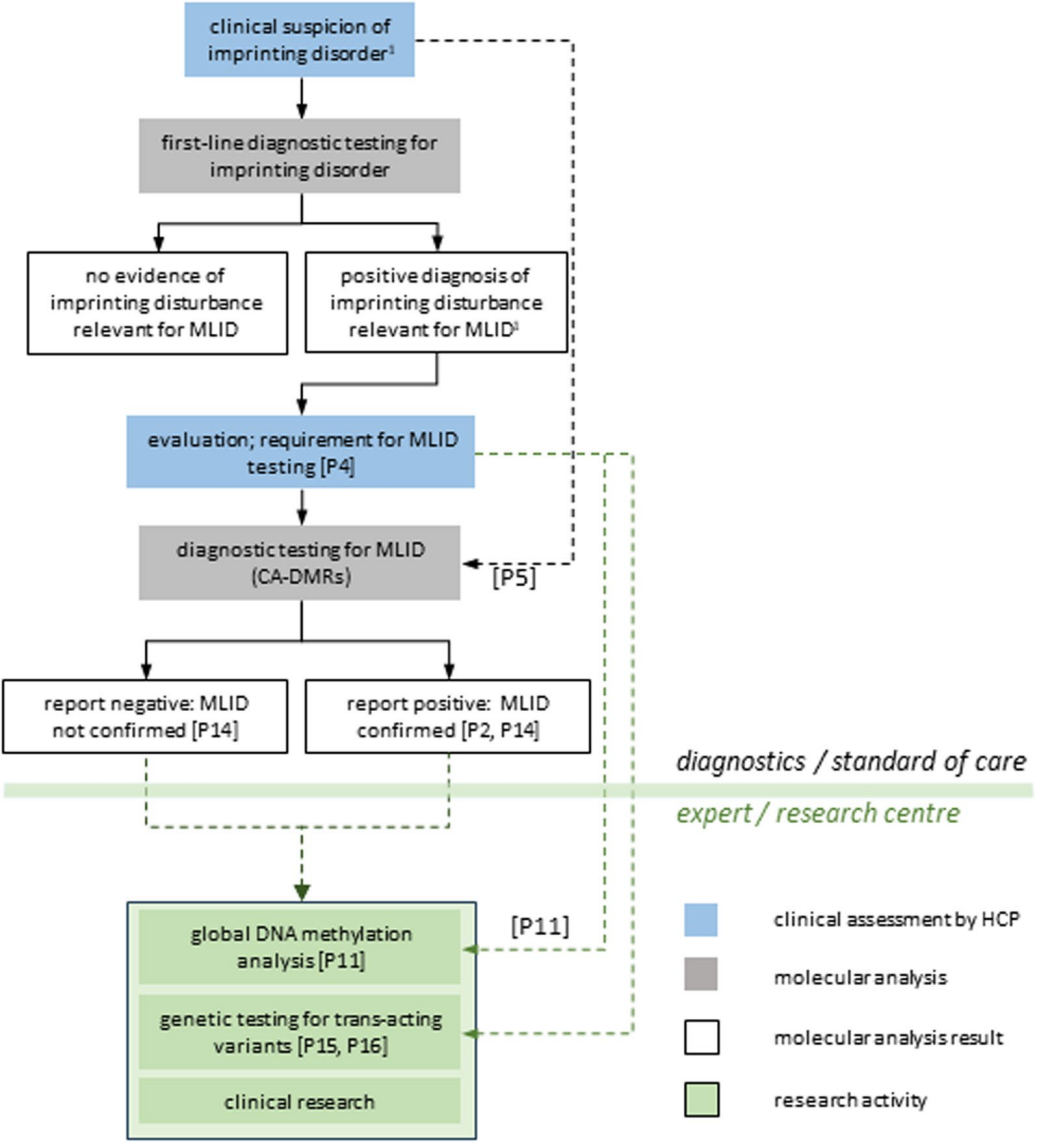
Flow chart for investigation of MLID in the context of imprinting disorder diagnosis. Solid black lines are followed when clinical suspicion of an imprinting disorder prompts testing as per consensus guidelines, and subsequent expert evaluation prompts second-line MLID testing (Sect. “Second-line testing for MLID following molecular diagnosis of an imprinting disorder”). When suspicion of MLID prompts expert referral for first-line testing of clinically associated differentially methylated regions (CA-DMRs), dashed black lines are normally followed. Dashed green lines are followed when diagnosis of MLID prompts further investigation in an expert centre or research setting, such as epigenome-wide DNA methylation analysis (Sect. “Molecular testing for MLID as a first-line test”), or genetic testing (Sect. “Genetic testing in MLID”). 1, Imprinting disturbances relevant for MLID include disorders and disturbances indicated in Table 2 and include any MLID detected during first-line multi-locus testing; P, proposition; HCP, health care professional

Individuals referred for molecular diagnosis of an imprinting disorder normally fulfil clinical criteria warranting referral, or have phenotypic features or a clinical history giving rise to a clinical suspicion of an imprinting disorder. However, individuals with MLID may have a phenotype closely consistent with a single imprinting disorder, with more than one imprinting disorder, or with no single imprinting disorder (Sect. "Multi-locus imprinting disturbance (MLID)"); or testing may be warranted by detection of MLID in a family history (Sect. "Genetic causes of MLID"). Because of its clinical heterogeneity, the diagnostic pathway for MLID can take variable forms. MLID diagnostic testing may be requested as a second-line test after a positive diagnosis of an imprinting disorder, or as first-line testing when explicitly warranted by the clinical or counselling situation (see also Sect. "The diagnostic decision tree for MLID"; Fig. 1).

#### Second-line testing for MLID following molecular diagnosis of an imprinting disorder

Second-line testing for MLID is additional testing that follows a positive molecular diagnosis of an imprinting disorder. MLID is chiefly associated with a subset of imprinting disorders, and a subset of molecular aetiologies of imprinting disorders (Sect. "Multi-locus imprinting disturbance (MLID)"; Table 2). MLID is a consideration for patients within these subsets, whereas patients outside these subsets have a very low likelihood of MLID.

After positive diagnosis of an imprinting disturbance for which MLID is a consideration, the patient information should be evaluated by an expert. If this evaluation identifies clinical and/or family features suggestive of MLID, or otherwise determines that MLID testing is warranted, the patient should undergo second-line testing for CA-DMRs. (Note that for the purposes of these guidelines, an “expert” is a healthcare professional, clinical or otherwise, with expertise in imprinting disorders/MLID as evidenced by professional qualifications such as clinical genetics training, research publications, or extensive experience in a relevant healthcare setting.)

#### Molecular testing for MLID as a first-line test

When expert clinical assessment gives rise to a suspicion of MLID, first-line diagnostic testing for MLID may be requested. Table 4 lists clinical features in patients or families which have prompted suspicion of MLID in published literature, but clinical translational research is necessary to establish the phenotypes that are most useful for this purpose. At present, DNA methylation analysis of CA-DMRs and NC-DMRs, genome-wide DNA methylation analysis, and genetic testing of causative genes are not recommended as first-line testing, unless in exceptional circumstances and under referral of a clinician expert in imprinting disorders.

**Table 4:** Atypical clinical features observed in individuals with MLID

Clinical feature	References
Sibling(s) with imprinting disorder, particularly with different imprinting disorders	[51, 53–56]
Overlapping clinical features of more than one imprinting disorder	[35–39, 80, 114﻿]
Atypical clinical features of imprinting disorder	[25, 30, 40–43]
Molecular diagnosis of BWS-LOM-IC2, SRS-LOM-IC1, TNDM, TS14, sporadic PHP1B	[4, 8, 9, 13, 14, 18, 19, 23, 25, 26, 32, 33, 35–37, 47, 49–51, 79, 85, 115]
Mother with probably pathogenic variant(s) in any maternal effect gene OR proband with pathogenic variants in trans-acting imprinting control gene	[34, 36, 37, 47, 49, 53, 54, 56–58, 116]
Parental history of large number (> 3) miscarriages	[34, 55, 58, 117﻿]
Conception involving ART	[10, 12, 15, 17, 66, 67]
	Imprinting Disorders (not MLID) in relation to ART: [68, 69, 118, 119]
Placental abnormalities such as placental mesenchymal dysplasia	[﻿^120^, ^121^]
Monozygotic twinning	[14, 55, 122, 123﻿]
Intellectual disability remaining undiagnosed after at least a complete work-up including CMA, CES/WGS, karyotyping, etc.	[124, 125]
Birth weight > 2SD above or below norm with any additional clinical feature	[35]
Current height or weight > 2SD above or below norm with any additional clinical feature	[43, 55]

*CMA* chromosome microarray; *CES* clinical exome sequencing; *WGS* whole genome sequencing; *LOM* loss-of-methylation; *IC* imprinting centre

### Prenatal testing for MLID

MLID testing is not recommended for prenatal testing, unless in exceptional circumstances and under referral of a clinician expert in imprinting disorders, because: (a) clinicians can request diagnostic testing of individual loci; (b) the DNA methylation of prenatal tissues is not always comparable with those of blood-derived DNA, so that control ranges for prenatal testing must be established using appropriate tissues; (c) prenatal samples can be technically harder to test than postnatal samples; (d) current commercial, diagnostically familiar MLID tests have fewer probes for each locus which are correspondingly more technically demanding to interpret; (e) interpretation of prenatal testing can be challenged by mosaicism [76–78]. Taking these factors into consideration, the consensus members were not in favour of recommending MLID testing in general diagnostic practice, while acknowledging that some expert centres may perform it and some situations may require it.

### Clinical value of a diagnosis of MLID

Expert assessment and counselling is required for patients diagnosed with MLID and their families, because MLID may have implications for clinical management, genetic counselling or both, and implications for the affected person or their wider family.

#### Implications for clinical management

A MLID diagnosis may alter the management or prognosis for the affected person. Some patients with MLID have clinical features of more than one imprinting disorder at presentation, and therefore their management takes account of this altered clinical situation and is informed by the molecular diagnosis. In some patients, the MLID diagnosis indicates a prognostic risk that additional clinical features might emerge in the future (for example, features of pseudohypoparathyroidism in a person with GNAS imprinting defect [79], or early puberty in a child with 14q32 imprinting defect [80]); surveillance for such potential complications should be included in their management plan. Some patients have clinical features that are atypical for any specific imprinting disorder but sufficiently suggestive of imprinting disturbance to prompt referral for MLID testing; in these cases, a positive diagnosis may give guidance for ongoing management and also has value in bringing an end to the diagnostic odyssey.

The general view of participants was that only CA-DMRs should be tested in a diagnostic setting, because the relevance of ‘non-clinical’ loci for clinical management is currently unknown, and their interpretation may be challenging for labs with limited experience in imprinting, which might result in unrequested, uninterpreted results being returned to clinicians or families.

#### Implications for genetic counselling

Investigation of MLID has counselling implications for the affected person and family members. Expert advice should be offered to the patient and their family, to help the family understand the testing performed and its results; to understand any changes in prognosis and potential changes in clinical management, with any specialist referrals required; to understand risks of recurrence and testing options for the wider family; and to make informed decisions about future reproductive choices.

If genetic testing is performed and identifies an underlying trans-acting pathogenic variant, implications for the family include transmission of the variant itself and, in the case of maternal effect variants (MEV, see Sect. "Maternal effect variants") a potentially separate risk of MLID and/or reproductive problems in family members. Detection of MLID (potentially including CA-DMRs and NC-DMRs) in family members of a proband may help assign pathogenicity to a detected variant. Family members undergoing cascade testing may require expert counselling to understand potential genetic causes, and clinical implications, including the potential for MLID in offspring or reproductive difficulties for parents.

### Use of the term MLID in the clinical setting

Many people with MLID have clear clinical features of one imprinting disorder (their ‘presenting’ disorder); in these situations, a dyadic term (such as BWS-MLID) may be used to indicate both the primary diagnosis and the additional molecular finding. Some patients’ clinical features are inconsistent with a single presentation or are atypical for any ‘classic’ imprinting disorder, and this clinical situation has occasionally been referred to as MLID (multi-locus imprinting disorder). MLID is a molecular designation and should not be used as a clinical designation for patients with MLID and atypical or idiosyncratic phenotypes. For these patients we propose the alternative term multi-locus imprinting syndrome (MLIS), which is related to but distinct from the term MLID, and which indicates the underlying causal relationship between patients whose phenotypic and epigenetic syndromic presentations may be distinct [81].

#### Propositions

P4. Diagnostic MLID testing following positive diagnosis of a relevant imprinting disorder should be offered on the basis of evaluation of the patient and family.

P5. MLID testing as a first-line referral should normally be offered on the basis of evaluation by an expert in imprinting disorders.

P6. MLID testing should not be offered as prenatal testing.

P7. Clinical management of MLID should take into consideration only methylation disturbance at loci directly associated with clinical imprinting disorders.

P8. Expert counselling is required for the family if MLID is identified.

#### Recommendation for Research

R2. There should be periodic review and update of the clinical indications for first-line MLID testing.

R3. There should be periodic evaluation of guidelines for laboratory testing of MLID, assessing whether any clinical indications or molecular diagnoses should directly trigger second-line MLID testing.

R4. There should be a periodic review of the clinical designations of individuals with MLID.

## Molecular diagnosis of MLID

### The distinction between multi-locus testing in diagnosis and testing for MLID

In ImpDis diagnostics, there is an important distinction between “testing for MLID” and “applying multi-locus testing” (MLT). For example, SRS is caused by genetic and epigenetic changes to chr11 and chr7, and overlapping phenotypes are associated with changes to imprinted loci on chr14, 20 and others. The SRS consensus guidelines [75] recommend MLT of chr7 and 11 as first-line diagnostic testing for SRS, and this is performed in many laboratories. Some laboratories perform MLT of all CA-DMRs in the diagnostic workup of SRS referrals, to maximise detection of all imprinting disturbances [72]. Thus, MLT is performed in first-line testing for some imprinting disorders and does not itself constitute MLID testing.

### The diagnostic decision tree for MLID

An outline decision tree is presented in Fig. 1. It presumes clear distinctions between.

- Diagnostic first-line testing, which may in some cases employ MLT;
- Diagnostic CA-DMR testing;
- Global MLID testing, which normally takes place in the context of research;
- Genetic testing, which would normally be performed and/or interpreted only in expert or research centres;
- Research.

Note that for the purposes of these guidelines, an expert centre is a clinical or laboratory centre with expertise in imprinting disorders/MLID. Expertise is evidenced by staff with publications and experience in the field, and/or quality assurance management, according to the DIN ISO 1589 which represents the international standard of quality management in genetic diagnostic testing. Additionally, the current turnover numbers of samples should be sufficient to cover all types of molecular disturbances observed in imprinting disorders.

The decision tree presumes the consensus statements for BWS, SRS and other imprinting disorders that are current at the time of writing, in which MLID testing is not recommended; these recommendations may change as the state of knowledge evolves.

The decision tree must be viewed in the context of local practice. Different countries and laboratories have variations in their legal and regulatory structures, processes for testing, costing models of testing, expectations for returning results to patients, access to clinical counselling and management, and relationships between diagnostic and research activity; any or all of these may modify the approach to MLID testing.

### Imprinted loci to be tested during MLID diagnosis

In research studies to date, the loci included in MLID testing have varied, depending on factors including the clinical features of the patient, additional features in the family history, the tests available in the testing centre, the funding for the testing performed, and the research-informed understanding of imprinted loci with clinical relevance [See Sect. "Multi-locus imprinting disturbance (MLID)"]. At the time of writing, the majority of diagnostic laboratories employ commercial MS-MLPA (methylation-specific multiplex ligation-dependent probe amplification [82] for ImpDis diagnosis [72], using kits containing probes for multiple CA-DMRs. As noted above (Sects. "A pragmatic diagnostic definition of MLID" and "Implications for clinical management"), only CA-DMRs should be tested in diagnostic settings.

For the specific purposes of MLID testing, it is not necessary to include all DMRs within each imprinted locus; this differs from diagnosis of some ImpDis where analysis of multiple DMRs can discriminate molecular aetiologies (e.g. PHP1B/iPPSD3 [83]).

Comprehensive MLID analysis (including ‘non-clinical’ loci) should be performed in expert centres and on a research basis, and not in routine diagnostics. Translational research analysis of MLID is essential to: (a) verify the DMRs to be included in clinical definition of MLID to optimise clinical diagnosis; (b) determine any further epimutations that should be analysed in cases that elude diagnosis using current testing, which uses a limited number of CpGs as a proxy for entire DMRs; (c) determine clinical correlations or genetic counselling relevance of loci currently understood as ‘non-clinical’; (d) clarify the relationship between MLID and causative trans-acting variants, including MEV (See Sect. "Genetic testing in MLID"). As stated above (Sect. "A pragmatic diagnostic definition of MLID") this research may in due course change the designated group of CA-DMRs included in clinical MLID testing.

### Tissues to be tested during MLID diagnosis

The vast majority of published studies, and the vast majority of standard-of-care tests, use blood-derived DNA. Normal control ranges and variability are well established for blood DNA compared with many other tissues. A small number of studies looking at multiple tissues in MLID patients have observed involvement of different loci and different methylation levels in different tissues (e.g. Refs. [84, 85]). If tissues other than blood are tested, normal methylation ranges must be established.

### Threshold levels of methylation disturbance to be achieved for positive MLID diagnosis

Diagnosis of many ImpDis is complicated by their inherent mosaicism. It is well recognised that some patients elude diagnosis because their methylation disturbance is not detectable in the tissue tested (normally blood) (reviewed in [86]). Currently there is no consensus on thresholds for a positive diagnosis, even in widely adopted methods like MS-MLPA.

Moreover, because assays vary in their biochemical bases and the DNA sequences analysed, the analytical protocols and analytical sensitivity correspondingly vary. In practice, laboratories pragmatically accept this inter-assay variation, and use inter-laboratory comparison to validate their testing and support their interpretation (such as [87]). National and international quality assessment schemes (such as those offered by EMQN [88]) formalise this cross-comparison and support harmonisation of testing and interpretation.

Different centres and health systems currently have different approaches to testing and reporting DNA methylation disturbance in patients. Diagnostic quality management guidelines recommend that methylation abnormality should be reported when methylation is outside lab-defined normal ranges, but the guidelines do not require specification of abnormal methylation indices. Some diagnostic labs with low sample throughput may have insufficient samples or experience to quantify and interpret methylation change. These laboratories must ensure suitable quality measures (e.g. analysis of control samples with different (epi)genotypes, participation in external quality assessment schemes) to demonstrate their capacity to identify the full spectrum of molecular alterations including mosaicism.

Methylation disturbance in MLID can vary between tissues, although in a given tissue the methylation appears stable across time (e.g. Refs. [8, 15, 85, 89]). This variation between tissues means that a numerical statement of methylation levels in a given tissue, such as blood, may suggest a biological precision that is at odds with the actual state of affairs in the affected person. As a result, diagnostic laboratories report methylation disturbance in different ways, e.g. referring to hypomethylation or hypermethylation, to partial or complete methylation changes, or to a numerical methylation level.

The degree of methylation change can provide circumstantial evidence towards the molecular aetiology of an imprinting disturbance and its clinical consequences; e.g. partial LOM at an imprinted locus suggests an elevated likelihood of MLID, compared with total LOM, which can be associated with a cis-acting genetic change (e.g. Refs. [90–92). In some ImpDis, partial LOM is associated with different or milder clinical features than complete LOM. For example, complete LOM at the *SNRPN* imprinted region is associated with AS, but patients with partial LOM do not have some of the cardinal features of AS and may elude clinical diagnosis [93, 94]; therefore, in patients where MLID involves SNRPN (e.g. Refs. [39, 42]), the level of methylation disturbance may indicate vigilance for different emerging clinical features.

Translational research is required, potentially in the format of a pilot quality assessment scheme, to determine (a) the loci to be included in testing, (b) the DMRs and CpGs that most reliably reflect the status of the whole; (c) the control methylation ranges for these regions; (d) thresholds for reporting abnormal methylation; (e) the statistical methods to be used to define normal versus abnormal methylation. These findings can then be incorporated into future recommendations for testing. Quality assessment for MLID testing may be established using the community’s experience of existing QM schemes for imprinting disorders.

### Laboratory reporting of MLID

Practices for genetic reporting vary between countries: for example, in some healthcare systems genetic reports are returned to patients, and in others to their referring clinicians. While laboratory reports should follow context-dependent practices, they should also follow international reporting guidelines, including information such as the patient demographics, results, interpretation of results, recommendations for counselling and management, and the validation status and limitations of the technique(s) used. Sequence variants should be interpreted using standards and guidelines for interpretation [95] and the internationally recognised standards for variant description should be employed [96]. Participants considered that laboratory reports should normally include the data in BOX 1.

### BOX 1: Data to be included in laboratory reports

- The rationale of testing, i.e., whether it is diagnostic or research-based;
- the imprinted loci tested (chromosome and genomic position). Internationally agreed nomenclature should be used instead of/in addition to local nomenclature. The clinical relevance of the loci should be clear (i.e., clinically associated/nonclinical loci);
- the tissue type of the sample tested (e.g., blood, saliva, etc.);
- the detection level of the assay used in the tissue tested;
- an indication of the level of methylation disturbance at each affected locus, including a statement of the laboratory’s policy on the level of precision in reporting DNA methylation (see below);

In the case of positive reports:

- Statement of the imprinting syndromes associated with the loci involved in MLID;
- Recommendation to refer to an expert centre;
- Recommendation to discuss the report with a healthcare professional expert in imprinting disorders; this is particularly important in health systems where genetic reports are directly returned to families;
- Reference to existing imprinting disorder consensus guidelines and recommendation to follow consensus guidelines where available;
- Recommendation to consider genetic testing, if relevant.

#### Propositions

P9. Multi-Locus Testing (MLT) is distinct from MLID testing.

P10. MLID testing should be performed with consideration of the decision pathway.

P11. Comprehensive MLID analysis (including ‘non-clinical’ loci) should be performed only in expert centres and on a research basis.

P12. MLID diagnosis should normally use blood-derived DNA. Other tissues should normally be tested in expert centres, and normal ranges should be determined.

P13. Methylation disturbance in MLID should be validated and quality-assured in the same way as for imprinting disorders.

P14. Diagnostic reports for MLID should follow international reporting guidelines

#### Recommendations for Research

R5. Comprehensive MLID analysis should include as many imprinted DMRs as fully as possible, including all DMRs in loci with multiple DMRs.

R6. Epigenotype-genotype–phenotype correlations should be collated to update the loci included in standard of care MLID testing for clinical and/or genetic counselling purposes.

R7. Trans-national and cross-platform comparison should be performed to assess whether / how DNA methylation disturbance in MLID should be reported numerically.

## Genetic testing in MLID

Many papers describe massively parallel sequencing (gene panel, clinical exome or whole genome) to detect trans-acting gene variants associated with MLID (reviewed in [45]). Screening for trans-acting SNVs has often employed trio sequencing of probands and both parents. While some health systems have implemented genetic testing for relevant cases in expert centres, potentially causative variants may also be identified through commercial exome testing in clinical genetics or reproductive medicine.

There is an urgent need for translational research and information sharing to address the current uncertainties about trans-acting genetic causes of MLID; but despite these uncertainties, there is a need for interim guidance on genetic testing in MLID.

### ZFP57

Biallelic *ZFP57* variants cause TNDM with MLID, with a characteristic DNA methylation signature of LOM of *PLAGL1*, *GRB10* and *PEG3*. Current ISPAD (International Society for Pediatric and Adolescent Diabetes) clinical practice consensus guidelines recommend *ZFP57* sequencing in individuals with TNDM when hypomethylation of *PLAGL1* is detected, without requiring MLID testing to confirm the distinctive DNA methylation signature [97].

### ZNF445

Pathogenic variants in *ZNF445* have been reported in a single individual with Temple syndrome (TS14) and MLID (TS14-MLID, [36]). Further research is required to assess the contribution of *ZNF445* to clinical MLID and thus its inclusion in diagnostic testing.

### Maternal effect variants

In the mothers of probands with MLID, pathogenic maternal effect variants (MEVs) have been reported in maternal effect genes, including *NLRP2*, *NLRP5*, *NLRP7*, *PADI6*, *OOEP* and *KHDC3L*. These are genes highly expressed from the maternal genome during oocyte maturation, whose products appear to play essential roles in the early embryo [59]. Unlike *ZFP57*, there seems to be no clear DNA methylation signature associated with these MEVs [45]﻿.

Biallelic MEVs have been identified in families with clear clinical evidence of MLID, such as siblings affected by MLID [53–56, 98]﻿. Because of this, a strong clinical suspicion of multiple affected offspring warrants familial genetic testing for maternal effect variants.

Offspring of a mother with biallelic penetrant MEV are at approaching 100% risk for experiencing MLID, which may involve imprinting disturbance of both CA- and NC-DMRs.

There is increasing evidence of patients with MLID whose mothers have heterozygous variants in maternal effect genes (reviewed in [45]); in these situations, it is particularly difficult to interpret variant pathogenicity and recurrence risk for mothers. Heterozygous MEV may be over-reported in research studies due to ascertainment bias. On the other hand, variant interpretation guidelines (particularly the ACMG guidelines [95] or constraint metrics such as those calculated in GnomAD [﻿^99^] are designed for penetrant Mendelian genes and rarely attribute pathogenicity to MEVs; therefore MEVs remain unreported by laboratories, which in turn means they remain uncollected in the literature and databases.

MEV would normally go undetected in standard genetic diagnostics because: (a) diagnostic laboratories do limited MLID testing, if any, therefore underestimate prevalence of MLID; (b) under-recognition of MLID leads to under-referral for genetic testing; (c) the poor fit of interpretation guidelines for MEVs leads to under-reporting of pathogenicity and consequent under-recognition of their role in MLID.

There is an urgent need for comprehensive trans-national research studies and data sharing to clarify the pathogenicity of MEVs and their role in MLID. Except for the most severe and clear clinical cases, MEV testing may currently be regarded as translational research, and therefore there should be a low threshold for recruiting families for research into MLID. Families where MEVs are not detected should be further investigated for the possibility to discover new genetic or environmental causes of MLID.

#### Propositions

P15. In the case of TNDM with LOM of *PLAGL1*, recessive *ZFP57* variants should be investigated and counselling given, following ISPAD guidelines.

P16. In families with siblings with MLID and/or reproductive history strongly suggestive of maternal effect variants, genetic testing of trans-acting genes should be considered.

#### Recommendation for research

R8. Cases of MLID should be collected trans-nationally to determine the penetrance and expressivity of MEV in MLID, and identify new causative genes, supporting the implementation of diagnostic testing as appropriate.

## Outlook and conclusion

MLID presents unique challenges for affected individuals, their families and their healthcare providers. It is phenotypically, epigenetically, and genetically intrinsically heterogeneous. Healthcare professionals need to be aware that the presence of MLID can modify a patient’s clinical presentation, management, long-term well-being, and familial risk of recurrence, in potentially unique ways.

The propositions presented here represent interim guidance on clinical and molecular diagnosis of MLID, based on published evidence and expert opinion. Because of the inherent variability of MLID, the guidelines emphasise the value of experts, including clinicians and allied healthcare professionals, for confident diagnosis, counselling, and care. It is hoped that these guidelines will encourage both a broadening of collaboration between local and expert practitioners (including international co-operation) and a deepening of clinical and molecular expertise across the community.

Despite the inherent heterogeneity of MLID, its connection with canonical imprinting disorders must be recognised. The authors hope that consensus guidelines for relevant imprinting disorders (including iPPSD3, BWS, and SRS) will in due course incorporate information and appropriate guidance about MLID.

The authors call for international, collaborative, basic and translational research to address many important questions that are currently unanswered. The required studies, as outlined in the Recommendations of this document, include investigation of the pathology, prevalence, and clinical features of MLID, the biology of maternal effect genes, and most importantly, the international collection of detailed clinical histories that is essential to guide confident diagnosis and underpin the development of clinical management guidelines. The authors recommend that within 3–5 years this expert group should re-convene to assess progress in research and update this guidance where appropriate.

### Supplementary Information

Additional file 1.

Additional file 2.

## Data Availability

No datasets were generated or analysed during the current study.

## Abbreviations

ACMG: The American College of Medical Genetics and Genomics
ART: Assisted reproductive technology
AS: Angelman syndrome
BWS: Beckwith-Wiedemann syndrome
CA: Clinically associated
CES: Clinical exome sequencing
CNV: Copy number variant
CPP: Central precocious puberty
DIN ISO: Deutsches Institut fur Normung; International Standardization Organization
DMR: Differentially methylated region
GOM: Gain-of-methylation
HIL: Hypomethylation of imprinted loci
IC: Imprinting centre
ImpDis: Imprinting disorders
ISPAD: International Society for Pediatric and Adolescent Diabetes
KOS14: Kagami–Ogata syndrome
LOM: Loss-of-methylation
MCBS: Mulchandani–Bhoj–Conlin syndrome
MEV: Maternal effect variants
MLID: Multi-locus imprinting disturbance
MLIS: Multi-locus imprinting syndrome
MLMD: Multi-locus methylation disturbance
MLT: Multi-locus testing
MS-MLPA: Methylation-specific multiplex ligation-dependent probe amplification
NC: Non-clinical
PAO: Patient advocacy organisation
PWS: Prader–Willi syndrome
SHFYNG: Schaaf–Yang syndrome
SNV: Single-nucleotide variant
SRS: Silver–Russell syndrome
TNDM: Transient neonatal diabetes mellitus
UPD: Uniparental disomy
WG: Working group
WGS: Whole genome sequencing
