# Supplementary material for: Multi-locus imprinting disturbance (MLID): interim joint statement for clinical and molecular diagnosis
Source: Clin Epigenetics. 2024 Aug 1;16:99. doi: 10.1186/s13148-024-01713-y (PMC11295890; doi:10.1186/s13148-024-01713-y)
Supplement: Supplementary file 1 — Additional file 1. [file 13148_2024_1713_MOESM1_ESM.docx]

| Supplementary Table 1 - Proposıtıons | |
| --- | --- |
| P1 | MLID is a molecular state of affairs where a person has DNA methylation disturbance involving multiple (non-contiguous) germline imprinted loci |
| P2 | For clinical reporting, MLID is designated as DNA methylation disturbances at ≥2 clinically associated (non-contiguous) DMRs |
| P3 | MLID is not constituted by imprinting disturbances of contiguous DMRs under co-ordinated control, or by apparent imprinting disturbances secondary to UPD or CNV |
| P4 | Diagnostic MLID testing following positive diagnosis of a relevant imprinting disorder should be offered on the basis of evaluation of the patient and family |
| P5 | MLID testing as a first-line referral should normally be offered on the basis of evaluation by an expert in imprinting disorders |
| P6 | MLID testing should not be offered as prenatal testing |
| P7 | Clinical management of MLID should take into consideration only methylation disturbance at loci directly associated with clinical imprinting disorders |
| P8 | Expert counselling is required for the family if MLID is identified |
| P9 | Multi-Locus Testing is distinct from MLID testing |
| P10 | MLID testing should be performed with consideration of the decision pathway |
| P11 | Comprehensive MLID analysis (including ‘non-clinical’ loci) should be performed only in expert centres and on a research basis |
| P12 | MLID diagnosis should normally use blood-derived DNA. Other tissues should normally be tested in expert centres, and normal ranges should be determined |
| P13 | Methylation disturbance in MLID should be validated and quality-assured in the same way as for imprinting disorders |
| P14 | Diagnostic reports for MLID should follow international reporting guidelines |
| P15 | In the case of TNDM with LOM of *PLAGL1*, recessive *ZFP57* variants should be investigated and counselling given, following ISPAD guidelines |
| P16 | In families with siblings with MLID and/or reproductive history strongly suggestive of maternal effect variants, genetic testing of trans-acting genes should be considered |

CNV, copy number variant; DMR, differentially methylated region; ISPAD, International Society for Pediatric and Adolescent Diabetes; LOM, loss-of-methylation; MLID, multi-locus imprinting disturbance; TNMD, transient neonatal diabetes mellitus; UPD, uniparental disomy;
