# Supplementary material for: Multi-locus imprinting disturbance (MLID): interim joint statement for clinical and molecular diagnosis
Source: Clin Epigenetics. 2024 Aug 1;16:99. doi: 10.1186/s13148-024-01713-y (PMC11295890; doi:10.1186/s13148-024-01713-y)
Supplement: Supplementary file 2 — Additional file 2. [file 13148_2024_1713_MOESM2_ESM.docx]

| Supplementary Table 2: RECOMMENDATIONS FOR RESEARCH | |
| --- | --- |
| R1 | The group of clinically-associated DMRs should be periodically reviewed and updated as necessary, based on ongoing clinical research |
| R2 | There should be periodic review and update of the clinical indications for first-line MLID testing |
| R3 | There should be periodic evaluation of guidelines for laboratory testing of MLID, assessing whether any clinical indications or molecular diagnoses should directly trigger second-line MLID testing |
| R4 | There should be a periodic review of the clinical designations of individuals with MLID |
| R5 | Comprehensive MLID analysis should include as many imprinted DMRs as fully as possible, including all DMRs in loci with multiple DMRs |
| R6 | Epigenotype-genotype-phenotype correlations should be collated to update the loci included in standard of care MLID testing for clinical and/or genetic counselling purposes |
| R7 | Trans-national and cross-platform comparison should be performed to assess whether / how DNA methylation disturbance in MLID should be reported numerically |
| R8 | Cases of MLID should be collected trans-nationally to determine the penetrance and expressivity of MEV in MLID, and identify new causative genes, supporting the implementation of diagnostic testing as appropriate |

DMR, differentially methylated region; MEV, maternal effect variants; MLID, multi-locus imprinting disturbance; UPD, uniparental disomy.
